# The Political Sociology of NICE: Investigating Pharmaceutical Cost‐Effectiveness Regulation in the UK

**DOI:** 10.1111/1467-9566.13878

**Published:** 2025-01-01

**Authors:** John Abraham, Gowree Balendran

**Affiliations:** ^1^ Department of Medical Education Brighton and Sussex Universities Medical School (BSMS) Brighton UK; ^2^ Department of Global Health and Social Medicine King's College London London UK

## Abstract

The National Institute for Health and Care Excellence (NICE) was established a quarter of a century ago in 1999 to regulate the cost‐effectiveness of pharmaceuticals (and other health technologies) for the NHS. Drawing on medical sociology theories of corporate bias, neoliberalism, pluralism/polycentricity and regulatory capture, the purpose of this article is to examine the applicability of those theories to NICE as a key regulatory agency in the UK health system. Based on approximately 7 years of documentary research, interviews with expert informants and observations of NICE‐related meetings, this paper focuses particularly on NICE's relationship with the interests of the pharmaceutical industry compared with other stakeholder interests at the meso‐organisational level. Consideration of the interaction between the UK Government and the pharmaceutical industry in relation to NICE is presented together with the analysis of revolving doors and conflicts of interest of NICE experts/advisors. The nature of policy changes over time (e.g. accelerated assessment pathways and industry fees for regulatory appraisals) and how they relate to the relevant stakeholder interests is also investigated. It is concluded that NICE is largely characterised by neoliberal corporate bias, though some elements of its organisation are also consistent with theories of capture, pluralism and polycentricity.

## 1 Introduction: The Research Need

**FIGURE 1:**
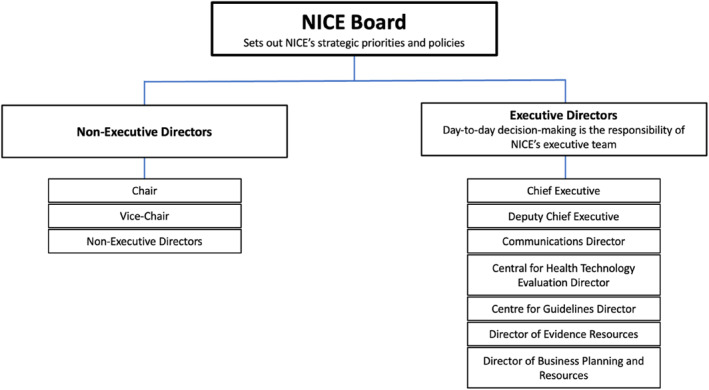
NICE's committee structure.

It is now a quarter of a century since NICE was installed by the UK Government in 1999; therefore, it is timely to consider from a sociological and political perspective what sort of institution it has been in that period. NICE is the National Institute for Health and Care Excellence, formerly and initially known as the National Institute for Clinical Excellence. In the UK, NICE regulates the cost‐effectiveness of health technologies—mostly pharmaceuticals (see Figure 1 for a representation of its committee structure). Its emergence may be seen as part of growing ‘evidentiality’ in governance of health‐care technology (Faulkner 2009). The fundamental aim of this paper is to consider the applicability of key political‐sociology theories of the regulatory state at the meso‐organisational level to NICE.

Worldwide, pharmaceutical products are regulated for ‘quality’ (adequate purity), ‘safety’ and ‘efficacy’ by government agencies. This legal duty falls to the Medicines and Health‐care products Regulatory Agency (MHRA) in the UK (Abraham and Davis 2020). If a drug is deemed to be safe, efficacious and of sufficient quality by the MHRA, then the manufacturer may place it on the market for ‘consumers’ (including the NHS) to ‘purchase’/prescribe. Such regulation excludes considerations of pricing or economic cost and the related government organisations (MHRA in the UK) are known as pharmaceutical *product* regulatory agencies, as distinct from *cost‐effectiveness* or *pricing* regulatory institutions. By contrast, NICE, as a *cost‐effectiveness* regulatory institution, produces (mandatory) ‘guidance’ on whether the NHS should purchase pharmaceutical products, which the MHRA has already approved on the market (Abraham 2009).

The approach used by NICE is cost–utility analysis (Rawlins 2012). Simply put, NICE asks: ‘Does a new drug approved by the MHRA offer greater value‐for‐money for the NHS than alternative (comparator health‐technology) interventions?’^1^ If NICE determines in the affirmative, then it recommends that NHS funds are expended on the (new) drug. Effectiveness is calculated in ‘quality‐adjusted life years’ (QALYs). A QALY is perfect health for 1 year. ‘Cost‐effectiveness’ is the additional cost per QALY gained over a comparator. NICE regards an incremental cost‐effectiveness ratio (ICER) of £20,000–£30,000 per QALY gained as being ‘cost‐effective’. Since ‘cost‐effectiveness’ is defined as a relative cost, built into the ICER is a sophisticated mechanism for pricing/expenditure control (or obversely, legitimation for high prices). As micro‐sociological analyses have shown, cost‐effectiveness assessment is not simple arithmetic but rather is characterised by techno‐scientific uncertainty, involving social and ethical judgements (Brown and Calnan 2013).

Within such uncertainty, it is important whose interests attain the benefit of the scientific doubt. The key stakeholder interests in contention are the commercial interests of the capitalist pharmaceutical industry and companies in maximising sales of their products to the NHS, the institutional interests of the NHS in spending its budget efficiently and the interests of consumers/patients in having access to the best health interventions available on the NHS. These interests may sometimes converge/coincide, but they may also diverge/conflict when pharmaceutical companies seek high prices to maximise profits, whereas the NHS wants to keep its costs down to maximise the range of health interventions it can offer consumers/patients (Gornall, Hoey, and Ozieranski 2016). Such conflicting interests are fully acknowledged by NICE (House of Commons Health Committee 2005).

Procedurally, after NICE's first appraisal committee meeting assessing a drug, an appraisal consultation document is published containing the committee's interim views. After consultation with stakeholders, including pharmaceutical manufacturers, medical/health professionals and patient organisations, NICE publishes a final appraisal determination, which becomes NICE's health technology appraisal guidance for the NHS.

On 1 July 2003, legal obligations were imposed by the Government on NHS trusts to provide funding within 3 months for all treatments recommended by NICE guidance to promote equitable access across the UK (Department of Health [DoH] 2003). That included the possibility that some treatments approved by NICE could be limited to only those patients meeting particular criteria. The introduction of NICE may seem like a cost‐containment policy development. However, drawing on information published on NICE's website, we analysed all 485 drug appraisals by NICE between April 1999 and March 2015, of which 84% resulted in positive guidance/approval from NICE for use on the NHS. Indeed, NHS expenditure on drugs increased by more than £1 billion annually after NICE was created (Hoey 2007; Regulatory Rapporteur 2008). Such observations underline the need for a meso‐level sociological investigation of NICE.

NICE is one of the world's first government regulatory agencies of pharmaceutical cost‐effectiveness and continues to enjoy an influential and prominent international reputation. Yet studies of NICE within medical sociology are rare and those employing a political‐sociology analysis are rarer. Notable exceptions are Brown and Calnan (2013), who conducted a micro‐sociological documentary analysis of NICE's final appraisal on four Alzheimer's drugs. Also, noteworthy, Gabe et al. (2012) and Main and Ozieranski (2021) have employed theories from political sociology to undertake micro‐sociological and meso‐organisational analysis of pharmaceutical cost‐effectiveness, respectively, but their focus is on another such government agency, namely PHARMAC in New Zealand, rather than NICE. Significant meso‐level political‐sociological analyses of the Polish Government's Agency of Health Technology Assessment, which was modelled on NICE, and its Hungarian counterpart, have also been completed by Ozieranski and King (2017) and Csanadi et al. (2019), respectively.

## 2 Political Sociology: Levels and the Research Problem

Political sociology may be regarded as having three levels: macro‐, meso‐ and micro‐. The Miliband–Poulantzas debate about the relationship between capitalism and the State (Barrow 2016) is an example of the macro‐level, whereas Gabe et al. (2012) exemplify micro‐level as they apply political‐sociology analysis to an individual drug controversy (Herceptin). Like Main and Ozieranski's (2021) study of PHARMAC, here, we present empirical research, and discuss theories relevant, at the meso‐level, that is, the sectoral (pharmaceutical health) and institutional (regulatory system) level.^2^

Perhaps the best known meso‐level political sociology of regulation is Bernstein's (1955) ‘life‐cycle’ theory of agency capture. On this view, regulatory agencies are established to protect the public interest following an industrial/sectoral disaster (e.g. mass drug injury). Accordingly, initially, the government agency zealously (and adversarially) regulates a capitalist industry at arm’s length in the ‘public interest’ (Mitnick 1980). However, following years of consultations/interactions with the regulated industry, including industry lobbying, revolving doors and conflicts of interests of agency personnel, the regulatory agency gradually becomes weakened and captured by the regulated industry. Thereafter, the agency regulates primarily in the commercial interests of industry, rather than the public interest, until another disaster occurs when the agency reverts to the beginning of the cycle.

‘Capture’ is a theory of regulatory agencies—the bureaucratic arm of the State. By contrast, corporate bias theory, developed by Abraham (1995) in relation to drug product regulatory agencies, examines the influence of the pharmaceutical industry on agencies directly as bureaucracies, but *also indirectly* via the executive (Cabinet/Presidential) and legislative (Parliamentary/Congressional) arms of the State. According to corporate bias theory, from the inception of agencies, industry enjoys privileged access to the regulatory process over other interests (e.g. patients/public health). A strong state and the industry jointly shape regulatory policy and decision‐making. Subsequently, in the context of neoliberal political developments, such as reductions in product safety/efficacy requirements and over 80% funding of the MHRA through competing for industry fees, Abraham revised his theory to ‘neoliberal corporate bias’ because neoliberalism is associated with minimisation of the regulatory state's independence from industry and with subjecting the state to tests of ‘market competition’ (Abraham and Lewis 2000; Davis and Abraham 2013).

An alternative to these perspectives is pluralist theory in which the state has no independent interests of its own and acts as a ‘referee’ in balancing/resolving the demands of various interest groups, none of whom hold a dominant or privileged position (Cawson 1986; McFarland 2004). The weak regulatory state of pluralism has some commonalities with theories of de‐centred and polycentric regulatory regimes in which a plurality of multiple non‐State actors take on regulatory functions and/or have authority over the regulatory agency (Black 2008).

These theoretical perspectives, which are not mutually exclusive, provide a framework with which to approach the research questions: what sort of meso‐level regulatory organisation has NICE been? Has it been captured? Is it best characterised by neoliberal corporate bias, pluralism or polycentricity?

## 3 Methodology

To answer these questions, we collected and analysed data from exhaustive documentary/archival literature, semi‐structured interviews and observations at NICE‐related meetings.

A systematic review of relevant social science, medical and pharmaceutical literature from 1987 was conducted together with the comprehensive analysis of NICE websites, NICE annual reports and corporate business plans, minutes of NICE Board/committee meetings, relevant NICE Evidence Review Group reports, UK Government web archive, DoH annual reports, Pharmaceutical Industry Competitiveness Task Force (PICTF) reports, minutes of Ministerial Industry Strategy Group (MISG) meetings, *Scrip* and newspaper archives and documented scientific meetings/conferences/speeches of relevant knowledgeable individuals. The literature review and documentary analysis have been ongoing from 2014 to 2024 but was predominantly conducted during 2014–2016.^3^ The literature search was cast widely. ‘NICE’ was a fundamental search term. Everything about NICE in the public domain up to 2017 (identified by all possible means, including search engines and citation follow‐up) was reviewed. From 2017, the literature and documentary database has been updated.

In exploring the applicability of regulatory theories, we collected documentary data on revolving doors and ‘conflicts‐of‐interest’ of NICE‐related personnel, as well as on industry fees (introduced from 2019) for NICE's appraisals. We focused on revolving doors of senior personnel (i) because they shape/direct agendas/policies/processes/public relations; and (ii) because such data were published, whereas detailed information about junior personnel is often unavailable. Revolving‐door data were collected between 2015 and 2021, ‘conflict‐of‐interest’ data from 2019 to 2020 and industry‐fee data between 2019 and 2024.

Potential interviewees were selected for their expert knowledge regarding the shaping, functions and/or regulatory decision‐making of NICE, typically identified by prior analysis of their publications and professional positions/experiences. That was the fundamental inclusion criterion rather than representativeness by institutional affiliation (e.g. academia/government/industry). Forty‐five interviews were sought, of whom 18 participated, including 7 key personnel from NICE, such as directors/committee chairs/members of appraisal committees, Evidence Review Groups, Clinical Guideline Development Groups and the NICE Technical Support Unit, as well as academics and representatives from industry and government. All interviews were audiotaped, transcribed and completed with ethical approval and informed consent. Also, 13 multi‐stakeholder meetings were observed, including NICE Fora, annual conferences and workshops—immediately after which detailed field notes were prepared. Interview and field note data were subsequently thematically coded and analysed to answer the research questions. When possible, triangulation between data sources was employed to strengthen the validity of our empirical findings (Guion 2002).

## 4 Establishing NICE: Privilege, Plurality or Polycentricity?

In 1994, the DoH and the Association of the British Pharmaceutical Industry (ABPI), whose membership includes transnational pharmaceutical firms headquartered in various countries around the world, jointly produced guidelines for the economic evaluation of pharmaceuticals to encourage the industry to give higher priority to cost‐effectiveness when funding drug research (Smee 2005). Five years before NICE was inaugurated, Dr Tom Walley, then Clinical Pharmacology Professor at Liverpool University, proposed an ‘independent’ UK institution ‘to act as an interface between clinicians, health service managers, the pharmaceutical industry and Government’, and provide ‘independent’ economic evaluations to guide drug‐purchasing decisions for cost‐effective prescribing. He elaborated:

This would be particularly welcomed by the industry and could be supported by offering a service whereby studies commissioned or conducted by industry could be validated for use by the NHS.

(Walley and Edwards 1994, 99)

According to Timmins, Rawlins, and Appleby (2016, 34), the methodology for technology appraisal by NICE evolved from recommendations of a DoH expert workshop on ‘Guidelines for Pharmaco‐economic Studies’ in 1997, which was a follow‐up to guidelines the department had outlined with industry in 1994. Workshop participants included leading figures from regional NHS Development and Evaluation Committees, DoH officials and several senior figures from the UK pharmaceutical industry. Its recommendations, completed in early 1998, were never published, so could never be scrutinised by the wider medical profession, patient organisations or citizens.

This close partnership between the Government and the pharmaceutical industry in the development of cost‐effectiveness assessment seems to be confirmed in 1996 by the Conservative Secretary of State for Health, Stephen Dorrell, who asserted:

[T]he (pharmaceutical) industry knows there will be no surprises, because our partnership is based on constant, constructive dialogue… In order to guarantee that the benefits of this are enjoyed by the UK economy, the Government is committed to ensuring that regulation of the sector is flexible and supportive.

(Corporate Watch 2003)

While pharmacoeconomics was valuable to industry for drug development strategies, pricing tactics and marketing, it might also encourage ‘medicines‐rationing’ that could be undesirable for the pharmaceutical industry (Walley and Edwards 1994, 94). When regionally funded evaluation committees judged medicines poor value for money, ‘purchasers’ resisted funding. Some GPs were also reluctant to prescribe expensive medicines. Consequently, access to some NHS treatments varied according to where patients lived (‘postcode lottery’), which conflicted with the NHS's fundamental principle to provide equal access to care according to clinical needs (Jones and Irvine 2003). Concerns about maintaining equitable distribution of health care escalated in 1998 when sildenafil (Viagra) was restricted on the NHS by the Government to avoid its huge cost, despite being an efficacious drug for which there was no oral substitute (Keith 2000; Parsons and Johnstone 2001; Stapleton 2018).

In this context, NICE was established by the New Labour Government on 26 February 1999 ostensibly as an ‘arm's length’ regulatory agency (McPherson and Speed 2022, 137; Rawlins 1999a). The New Labour Government sought to preserve a strong partnership with the pharmaceutical industry, as a former special advisor in the Policy Unit at 10 Downing Street confirmed when interviewed:

The primary purpose [in establishing NICE] was to get greater equity in terms of access but in doing that, there would have been no point in setting it up in such a way that it drove a very successful and strong British industry out of the country. So in that sense, of course, you've got to balance interests.^4^

It did not take long for the Government's balancing of interests to become apparent. In October 1999, after NICE issued its first verdict *against* the drug zanamivir (Relenza), senior executives from GlaxoWellcome, SmithKlineBeecham and AstraZeneca contacted the Prime Minister protesting that the decision ‘had potentially devastating consequences’ for their companies (Anon 1999). In response, within a month, the Government and pharmaceutical industry formalised an alliance, the Pharmaceutical Industry Competitiveness Task Force (PICTF), to provide ‘a structured, action‐orientated platform for effective dialogue between the Government and the pharmaceutical industry’ whereby a ‘policy of “no surprises” will be delivered more effectively by a much stronger and more senior ongoing relationship between the Government and the industry’ (PICTF 2001, 2–7). In November 2000, NICE reversed its guidance on Relenza ostensibly after the manufacturer submitted more data, though many NHS medical experts questioned if the scientific evidence had really changed (House of Commons Health Committee 2002). Prime Minister Blair characterised PICTF as emblematic of his commitment to ensure:

….a supportive regulatory framework, and an environment conducive to the research needed to discover cures of the 21st century. A key feature in maintaining the UK's attractiveness will be effective partnership at the highest levels between Government and industry.

(PICTF 2001, 1)

PICTF focused on bringing Government ministers and senior industry leaders together ‘to identify and report to the Prime Minister on steps that may need to be taken to retain and strengthen the competitiveness of the UK business environment for the innovative pharmaceutical industry’ (PICTF 2001, 5). Other stakeholders in health care were excluded from PICTF (Corporate Watch 2003; PICTF 2001). It provided a platform for privileged access by the pharmaceutical industry to the Government to ‘discuss fully and jointly the detail of the industry's concerns about how NICE operates’, thereby addressing ‘broader impacts on market access and the resulting competitiveness of the UK as a global player’ (PICTF 2001, 7). PICTF and its successor the Ministerial Industry Strategy Group (MISG), were established to promote the interests of the British pharmaceutical industry (MISG 2002). As one informant put it: ‘it wasn't ever intended just to be a rationing tool…it was always envisaged that it would have a broader role as well’.^4^ Evidently, the state created NICE to fulfil its own broader economic and political ambitions, including the commercial interests of the pharmaceutical industry. Thereafter, the industry was permitted to shape government policy for NICE through PICTF and MISG. Indeed, at MISG's first meeting, the timing and topic selection for NICE appraisals were discussed with the ABPI, and a draft consultation document was shared with them for comments before consultation with other stakeholders (MISG 2001).

Moreover, on 26 February 1999, NICE was instructed by the Secretary of State to appoint the Partners Council, which was to advise the NICE Executive Board on its work programme, methods and policy/strategy (DoH 1999; NICE 2000; Rawlins 1999b). The Partners Council membership was drawn from medical professional bodies, patient associations and relevant health‐care industries, including the ABPI (NICE 2000; NICE Annual Report 2000/2001a). The NICE Corporate Plan (2000–2003, 13) describes the Partners Council as ‘a rich source of advice on every aspect of our work, both from individual members and collectively’. Hence, the pharmaceutical industry was consulted intensively on the methods and work programme of NICE from the outset together with opportunities to influence NICE policies (NICE Annual Report 2000/2001b).

This is further evidence that the interests of the pharmaceutical industry were integrated into NICE's objectives from its inception, though unlike PICTF, the industry was not the only stakeholder involved. The Partners Council embraced a plurality of interests and there is no evidence to suggest that it prioritised the interests of the industry over other stakeholders. However, it should be noted that the presence of the pharmaceutical industry within the Partners Council meant that the industry (albeit along with other stakeholders) was permitted to advise NICE about the development of appraisal processes and methods for judging the industry's own products—an institutional conflict of interest.

On 19 May 2010, the Partners Council was disbanded because its functions were incorporated into other committees/processes within NICE (NICE Annual Report 2009/2010; NICE 2010).^5^ One such process is the NICE/ABPI Industry Council, which became effective from 2013 to provide ‘a framework within which the ABPI and NICE can address strategic issues of mutual interest’ (NICE/ABPI 2013, 1–4). This Council is co‐chaired by the ABPI President and NICE Chairman, consisting of Board members exclusively from those two organisations (NICE/ABPI 2013). It meets biannually to discuss mutual objectives, supplemented by quarterly meetings ‘and through other, mutually agreed, opportunities to address issues as required’, indicating that frequent consultation exists at senior levels between NICE and the ABPI (NICE/ABPI 2013, 4).

The balance of evidence on the establishment of NICE suggests mostly a privileged partnership‐approach with industry, rather than pluralism or polycentricity, though the latter both feature to a lesser extent.

## 5 Revolving Doors

One empirical indicator of capture theory is a revolving door of personnel between the regulatory agency and regulated industry. In corporate bias theory, the revolving door is also an important indicator but is extended to include executive and/or legislative officials beyond the bureaucracy (regulatory agency). Hence, this section explores revolving doors between senior personnel/policy‐makers at/for NICE and the pharmaceutical industry. This is also relevant to pluralism by default because less capture/corporate bias *may* imply more pluralism. Neither a revolving door nor a financial conflict of interest of an individual regulator/expert necessarily means that an individual has regulated/assessed in the interests of industry over public health. Nor does it imply that the individual has behaved improperly in any way. Capture and corporate bias are theories of institutions/collectivities, not individuals. Fundamentally interested in the former, we choose not to name individuals. Nonetheless, such theories do assert that the greater the extent of revolving doors and conflicts of interest across a regulatory system, then the greater the likelihood of capture and/or corporate bias of the regulatory agency.

Starting with the most senior, the Secretary of State (2010–2012) subsequently joined the pharmaceutical firm, Roche, as an advisor on pharmaceutical supply/pricing (Iacobucci 2015; Mason 2015; Peedell 2011). Next, the leader of the team, who set up NICE for the Government and developed its policy framework, later moved on to become an advisor to the pharmaceutical industry on health technology assessment (Pharmaphorum 2013; PR Newswire 2013).

Within a year of retirement from NICE, the Chairman of NICE, from 1999 to 2013, accepted an appointment to the Board of Directors at Intracellular Therapies (a bio‐pharmaceutical company) for a 3‐year term ending in 2017 (Jack 2013; United States Securities and Exchange Commission [USSEC] 2014b). That position at the Board of Directors was associated with financial remuneration and rewards (USSEC 2014b). This former NICE Chairman resigned from that Board in November 2014 and was appointed MHRA Chairman from 1 December 2014 (USSEC 2014a).

Revolving doors are one form of a wider phenomenon that Wedel (2009) refers to as ‘coincidences of interests’ pursued by elite government officials. For example, the Vice Chairman of NICE from 1999 to 2003, who was ‘instrumental in advising NICE’ on methodologies for cost‐effectiveness evaluation, was also Vice Chairman of the ABPI‐funded Office of Health Economics (OHE) from 1997 to 2001 (Buxton 2006, 1136; NICE Annual Report 2003/2004). Hence, for 2 years (1999–2001), this official held senior positions simultaneously at NICE and the pro‐industry/industry‐funded ‘think‐tank’, OHE. In February 2007, s/he was appointed Chairman of NICE's Research and Development (R&D) Advisory Committee and served until 2010 (NICE Annual Report 2006/2007). In January 2014, OHE announced that s/he had stepped down as Chair of OHE Editorial and Policy Boards after serving in these positions for 16 and 12 years, respectively (OHE 2013; OHE News 2014). Evidently, this official was simultaneously Chairman of OHE's Policy Board and Chairman of NICE's R&D Advisory Committee for 3 years.

Previous employment in industry also appears to be an attractive qualification for appointment to senior positions at NICE. The Centre for Health Technology Evaluation at NICE develops all technology‐appraisal guidance and is responsible for NICE's Scientific Advice programme, R&D programmes and Patient Access Scheme Liaison Unit (NICE 2016a). At the Centre, the most senior employees involved in technology appraisal came from the pharmaceutical industry. For example, the former Director of the Centre was previously employed in pharmaceutical R&D at GlaxoSmithKline for 8 years (NICE 2016c). S/he was also President of Health Technology Assessment International and a Scientific Committee member of the Innovative Medicines Initiative (IMI). The IMI research agenda is set by the pharmaceutical industry at the EU level—‘Industry steers while the European taxpayer fuels the venture’ (Hodgson 2008, 717). On 12 April 2018, the revolving door turned full cycle when s/he resigned from NICE after 17 years and was appointed as ABPI Chief Scientific Officer (ABPI News 2018).

A subsequent Director of this Centre was previously the Director of the Technology Appraisal Programme at NICE. This official set up the single technology appraisal programme in 2006 to expedite NICE appraisals (NICE 2006). S/he was previously employed at the pharmaceutical firm, Eli Lilly, for 4 years, working on pharmaceutical sales (NICE 2021).^6^

The Programme Director for Scientific Affairs at the Centre oversees NICE Scientific Advice, NICE Office for Market Access and the Science Policy and Research programme (Health Network Communications 2016). One such Director previously worked for 20 years in the pharmaceutical, chemical and contract laboratory industries before joining NICE (Health Network Communications 2016). NICE Scientific Advice provides fee‐for‐service consultancy to pharmaceutical companies and advises on how product development plans could generate the right health technology assessment evidence for future submission to NICE (NICE Annual Review 2014/2015).

Finally on NICE's ‘revolving doors’, the first Communications Director at NICE was Head of National Health‐care Development for AstraZeneca Pharmaceuticals before joining NICE in 1999 (National Prescribing Centre 2001; The Pharmaletter 1999). Hence, NICE's portrayal to the media and wider public was run by someone who came from the pharmaceutical industry.

Our research focused on relatively senior officials, but this might underestimate the extent of the phenomenon within NICE because similar research on Polish and Hungarian Health Technology Assessment agencies suggests that revolving doors are also significant among *mid‐level* government officials (Ozieranski and King 2016).

## 6 ‘Independence’ and ‘Conflicts of Interest’

Another important meso‐level indicator relevant to theories of capture and corporate bias is the independence of regulators and their expert/specialist advisors from the regulated industry. The NICE Technology Appraisal Committee (TAC) reaches its conclusions about the cost‐effectiveness of pharmaceuticals in a single technology appraisal by considering evidence submitted by the manufacturer, the evidence review group report, stakeholders' comments and advice from clinical specialists. From October 2009, companies with products undergoing appraisal became entitled to attend TAC meetings and receive a debrief from NICE (Office for Life Sciences 2010). This goes beyond pluralist engagement with stakeholders to another institutional conflict of interest.

NICE draws on ‘clinical specialists’ to assist it with appraisals. It defines them as follows:

Clinical specialists act as expert witnesses to the Appraisal Committee. They have specialist expertise and personal knowledge of the use of the technology and other treatments for the condition. They often have insights not typically available in published literature.

(NICE 2009, 58)

Previous research has found that financial links with pharmaceutical companies are prevalent among patient organisations contributing to appraisals at NICE (Mandeville et al. 2019; also see Ozieranski et al. 2021). However, such financial interests among clinical specialists and TAC members contributing to appraisals at NICE have never before been investigated. Regarding ‘interests’, in 2008, NICE policy stated:

A personal pecuniary interest involves a current personal payment, which may either relate to the manufacturer or owner of a product being evaluated in which case it is regarded as ‘specific’ or to the industry/sector from which the product comes, in which case it is regarded as ‘non‐specific’.

(NICE 2008a, 7)

Thus, ‘specific’ interests are distinctly and officially defined as ‘funding from manufacturer(s) of a technology under appraisal or competitor products’ (Mandeville et al. 2019, 1; NICE 2008a). NICE policy on declaring interests is leniently limited to the 12‐month period preceding the appraisal (NICE 2008a; NICE 2014). Applying NICE's (2008a) policy criteria, we examined all declarations of interests made by clinical specialists and committee members who contributed to appraisals conducted by NICE's Technology Appraisal Committee A (TAC A) between October 2009 and December 2015 (75 months) to systematically identify (i) the prevalence of ‘specific’ interests among clinical specialists and committee members contributing to TAC A appraisals; and (ii) the extent to which ‘specific’ interests were declared.

Our sampling of TAC A, rather than any of NICE's other Technology Appraisal Committees (B, C, or D), is, in effect, random. There is no reason to believe that results would be significantly different had we selected other TACs. Furthermore, the period selected is a very substantial proportion (25%) of, and falls almost exactly in the middle of, NICE's 25‐year institutional life to date, so is least likely to favour any one regulatory theory.^7^ Declarations of interests were identified from minutes of TAC A meetings on the NICE website. Between October 2009 and December 2015, TAC A held 54 meetings, but minutes were only available for 50 (Table 1). In those 50 meetings, there were 84 appraisals and 126 separate occasions on which a clinical specialist contributed to an appraisal. We counted each such occasion as a separate event. There were altogether 70 declarations of interests from clinical specialists (56%). On 20 occasions, clinical specialists were nominated by manufacturers whose products were being appraised (Table 1).

**TABLE 1:** Clinical specialists' conflicts of interests.

		Months				Subtotals	Number of TAC A committee meetings in that year	Number of appraisals
Year		January–March	April–June	July–September	October–December			
2009	C				0	**0**	2 (Oct/Nov)	3
	F				0	**0**		
	N				0	**0**		
2010	C	11	5	2	3	**21**	10 (Jan/Feb/Mar/Apr/May/Jun/Jul/Aug/Oct/Nov)	15
	F	9	3	0	3	**15**		
	N	0	0	0	0	**0**		
2011	C	3	2	12	3	**20**	7 (Jan/Feb/May/Jul/Aug/September/Nov)	12
	F	2	0	8	2	**12**		
	N	0	0	1	1	**2**		
2012	C	2	5	8	5	**20**	8 (Jan/Feb/Mar/Apr/May/Jul/Sept/Nov)	10
	F	2	3	8	3	**16**		
	N	0	2	2	1	**5**		
2013	C	11	3	4	No meetings/minutes	**18**	6 (Jan/Feb/Mar/Apr/Jul/Sept)	12
	F	6	0	3		**9**		
	N	0	0	0		**0**		
2014	C	2	3	9	4	**18**	7 (Jan/Jun/Jul/Aug/Sept/Oct/Nov)	14
	F	2	0	3	0	**5**		
	N	1	0	1	1	**3**		
2015	C	8	10	9	2	**29**	10 (Jan/Feb/Mar/Apr/May/Jun/Jul/Aug/Sept/Oct)	18
	F	4	5	3	1	**13**		
	N	3	5	1	1	**10**		
Totals	C					**126**	**50**	**84**
	F					**70**		
	N					**20**		

*Note:* C = Number of clinical specialists attending appraisals; F = Number of clinical specialists declaring financial interests; N = Number of clinical specialists nominated by drug manufacturers.

Each occasion on which a TAC A committee member contributed to an appraisal was also counted as a separate event. Hence, there were 1698 contributions from committee members between October 2009 and December 2015 and 222 (13%) declarations of interests (Table 2).

**TABLE 2:** TAC A members' conflicts of interests.

Year		Months				Subtotals	Number of TAC A committee meetings in that year	Number of appraisals
		January–March	April–June	July–September	October–December			
2009	A				66	**66**	2 (Oct/Nov)	3
	F				8	**8**		
	E				5	**5**		
2010	A	129	80	39	64	**312**	10 (Jan/Feb/Mar/Apr/May/Jun/Jul/Aug/Oct/Nov)	15
	F	18	20	7	9	**54**		
	E	8	7	3	2	**20**		
2011	A	38	60	112	19	**229**	7 (Jan/Feb/May/Jul/Aug/September/Nov)	12
	F	8	9	22	6	**45**		
	E	0	1	11	4	**16**		
2012	A	63	46	68	46	**223**	8 (Jan/Feb/Mar/Apr/May/Jul/Sept/Nov)	10
	F	13	8	10	5	**36**		
	E	5	3	3	2	**13**		
2013	A	169	61	61	No meetings/minutes	**291**	6 (Jan/Feb/Mar/Apr/Jul/Sept)	12
	F	12	2	4		**18**		
	E	5	0	3		**8**		
2014	A	24	47	99	111	**281**	7 (Jan/Jun/Jul/Aug/Sept/Oct/Nov)	14
	F	5	1	12	14	**32**		
	E	2	1	9	10	**22**		
2015	A	90	91	79	36	**296**	10 (Jan/Feb/Mar/Apr/May/Jun/Jul/Aug/Sept/Oct)	18
	F	9	11	8	1	**29**		
	E	6	11	4	1	**22**		
Totals	A					**1698**	**50**	**84**
	F					**222**		
	E					**106**		

*Note:* A = Number of TAC A members attending appraisals; E = Number of TAC A members excluded from appraisals; F = Number of TAC A members declaring financial interests.

From 2014, NICE defined ‘specific interests’ more narrowly to ‘the matter under discussion’ with additional flexibility for advisory committees in how the latter could be interpreted:

An interest is ‘specific’ if it refers directly to the matter under discussion. An interest is ‘non‐specific’ if it does not refer directly to the matter under discussion. For the purpose of applying this Code to committee members, ‘the matter under discussion’ will be defined…before a new topic is introduced.

(NICE 2014, 4–5)

The new definition implied that payment from manufacturers of the product or comparator was no longer a ‘specific’ interest unless it was for the product being evaluated in that appraisal. This relaxed NICE's criteria for ensuring transparency in declarations of interests and the ‘independence’ of experts from industry. Arguably that policy change in itself prioritised industry interests over public interest in maximum transparency. In our analysis, all personal payments received from manufacturers of products or competitor/comparator products (even if not directly related to products being evaluated) were considered to be ‘specific’ interests in accordance with the broader criteria specified by NICE policy in 2008.

NICE's 2008 policy unequivocally stated that individuals with personal ‘specific’ pecuniary interests should ‘declare and withdraw’ (NICE 2008a, 14). Nevertheless, we found that on several occasions, clinical specialists and committee members, who received personal payments from the manufacturer of the product or comparator unrelated to the matter under consideration (e.g. for a different product developed by the same manufacturer), did not declare ‘specific’ interests but, instead, incorrectly declared ‘non‐specific’ interests that were accepted by the appraisal committee and published without correction in meeting minutes.

Moreover, we discovered inconsistencies in how involvement with manufacturers of the technology or comparator was declared by different individuals or even by the same individual at different times (sometimes as a ‘specific’ pecuniary interest, at others as ‘non‐specific’). Personal ‘specific’ pecuniary interests appear to have been incorrectly classified as ‘non‐specific’ on numerous occasions. Some clinical specialists declared a ‘non‐specific’ pecuniary interest when funding was received from several manufacturers, including the manufacturer of the product being appraised, which is misleading because multiple ‘specific’ interests do not equate to a ‘non‐specific’ interest. At least one clinical specialist, who was nominated by the manufacturer of a product being appraised and had ongoing collaboration with the manufacturer, did not declare any ‘specific’ interests at the appraisal. Notwithstanding these inaccuracies, not a single clinical specialist was prevented from participating in an appraisal, even when they declared personal ‘specific’ financial interests involving the manufacturers of technologies under appraisal. To a lesser extent, inconsistent declarations of interest were found among appraisal committee members. Further analysis shows the proportion of declared interests that were ‘specific’ interests (Table 3).

**TABLE 3:** Declarations of personal pecuniary interest by clinical specialists and TAC A members.

Year	Number attending	Number of declarations of pecuniary interest	Number of declarations of personal specific pecuniary interests	Number of undisclosed (incorrectly classified) personal specific pecuniary interests	Prevalence of personal specific pecuniary interests
	Clinical specialists (TAC A members)	Clinical specialists (TAC A members)	Clinical specialists (TAC A members)	Clinical specialists (TAC A members)	Clinical specialists (TAC A members)
2009	0 (66)	0 (8)	0 (5)	0 (0)	0 (5)
2010	21 (312)	15 (54)	2 (19)	12 (9)	14 (28)
2011	20 (229)	12 (45)	4 (22)	8 (8)	12 (30)
2012	20 (223)	16 (36)	12 (11)	4 (13)	16 (24)
2013	18 (291)	9 (18)	0 (8)	9 (3)	9 (11)
2014	18 (281)	5 (32)	3 (21)	1 (1)	4 (22)
2015	29 (296)	13 (29)	9 (23)	4 (2)	13 (25)
**Total**	**126 (1698)**	**70 (222)**	**30 (109)**	**38 (36)**	**68 (145)**

Our findings indicate that financial conflicts of interest among the experts advising NICE on product appraisals are considerable. In over half (56%) of the 126 occasions on which clinical specialists contributed to appraisals, 70 of them saw financial conflicts of interest declared, whereas in over an eighth (13%) of 1698 occasions on which appraisal committee members contributed, 222 saw such conflicts declared. Our data also suggest that financial relationships with industry among individuals contributing to appraisals at NICE have been underestimated in the minutes of appraisal committee meetings published by NICE. Official minutes show that 42.9% of declarations by clinical specialists and 49.3% of declarations by TAC A committee members were for personal ‘specific’ financial interests, but our research indicates that it may be as high as 97% (68 of 70) and 65% (145 of 222), respectively.

## 7 Minimising the ‘Independent’ Regulatory State

Within NICE's original evidence assessment phase, submissions on cost‐effectiveness would be invited from the product's manufacturer (NICE 2001; Buxton 2001). NICE would then commission an independent academic group to produce a technology assessment report based on the systematic review of clinical and economic evidence about the product, together with a critical evaluation of the manufacturer's submission. Between 1999 and 2023, NICE's technology appraisal processes have become progressively faster by requiring less stringent evidence assessment and full funding of appraisals by pharmaceutical manufacturers. In the early 2000s, delays in prescribing expensive new drugs while appraisals by NICE were in progress came to be known as ‘NICE blight’ (Jones and Irvine 2003). Some manufacturers, patients and health‐care decision‐makers protested against ‘NICE blight’ and it became ‘a political imperative’ to speed up the appraisal process (Kaltenthaler et al. 2011, 1158). In September 2005, the NICE Board was required by Government to approve proposals to introduce single technology appraisal for new drugs (Chalmers 2007; NICE 2005, 2006, 2009). Thereafter, the older original assessment process became known as ‘multiple technology appraisal’.

Acceleration of NICE's drug appraisal is certainly in drug companies' commercial interests because it gives them sooner access to NHS markets. It may sometimes also be in the interests of patients with particular diseases provided that NICE's accelerated decision accurately reflects the reality of the cost‐effectiveness of the new drugs relative to alternative NHS treatments already available. Whether such acceleration is in the wider interests of the NHS or public health depends on whether the fast tracking leads to decisions which unrealistically overestimate the cost‐effectiveness of ‘promissory’ pharmaceuticals (Hedgecoe and Martin 2003).

In single technology appraisal, only evidence submitted by the pharmaceutical manufacturer is evaluated by an independent academic group, the evidence review group. Unlike in multiple technology appraisal, the evidence review group does not undertake an *independent* systematic review, and so it is only able to critique the manufacturer's submission. While single technology appraisal is quicker than multiple technology appraisal, the former relies entirely on evidence of cost‐effectiveness submitted by the manufacturer of the drug being appraised (Barbieri, Hawkins, and Sculpher 2008). Thus, in introducing single technology appraisal, NICE withdrew from some elements of its regulation of pharmaceutical cost‐effectiveness and allowed industry assessments to fill the resulting gap. This type of accelerated pathway seems likely to increase the risk of exaggerating cost‐effectiveness assessments, which are suboptimal for the interests of public health—a risk raised recently regarding overdependence on industry data/analysis underpinning NICE's approval of the expensive hypercholesterolaemia drug, inclisiran (Leqvio) (Michaels 2023).

From 2017, NICE introduced a new fast track appraisal process. Apparently, this offers a ‘lighter touch’ appraisal (compared with single technology appraisal or multiple technology appraisal) and applies to technologies deemed to offer exceptional value for money by costing less than £10,000 per QALY gained (NICE 2017a, 2). In fast track appraisal, evidence assessment is conducted by a team comprising the evidence review group, NICE technical staff and members of the TAC, offering ‘greater opportunity’ to seek clarification from manufacturers about their submissions (NICE 2017b, 6). This is intended to shorten the appraisal process by facilitating more work to be completed before the appraisal committee meeting, thereby maximising early decision‐making after the first appraisal committee meeting, and enabling the appraisal committee to cope with projected increases in appraisals without growing capacity. Nonetheless, fast track appraisal not only relies entirely on evidence submitted by drug manufacturers (like single technology appraisal) but also increases opportunities for direct consultation between manufacturers and appraisal committee members *prior to* the appraisal.

NICE guidance is based on the best available evidence, which ‘may not, however, be very good and is rarely complete’ (Rawlins and Culyer 2004, 224). Hence, the TAC has to make science‐based value judgements about the extent to which decisions should be influenced by incomplete evidence (Rawlins, Barnett, and Stevens 2010; NICE 2008b). The TAC also makes social value judgements regarding efficient allocation of resources and appropriate health‐care delivery for the NHS (Sculpher, Drummond, and O'Brien 2001). Ultimately, all NICE value judgements have been decided by the expertise and experience of the NICE Board of Directors, NICE advisory committees and the NICE Citizens Council—a panel of 30 members of the public reflecting UK demographics, though superseded by ‘NICE listens’ since 2021 (Rawlins and Culyer 2004; NICE 2016b; McPherson and Speed 2022).

In addition to cost per QALY gained, NICE also considers ‘the innovative nature’ of new technologies when making decisions. Notably, pharmaceutical industry employees, who were industry members of the steering group that updated NICE's guide to methods of technology appraisal, have advocated that ‘broader definitions of value, including how innovation is rewarded’ should be adopted by NICE's appraisal committees to ensure reasonable return to industry on investments (Earnshaw and Lewis 2008, 725–727).

In 2018, NICE reported in the council that it would be ‘challenged with managing a budget deficit in 2019/2020’ because its funding from the DoH ‘remained static’ and would therefore need to introduce charges for appraisals from April 2019 to raise about £9M annually (NICE‐Industry Council 2018, 2). From April 2019, NICE's pharmaceutical appraisals are funded by fees from drug companies, initially amounting to £3.6M (5.2%) of the agency's income in 2019–2020. This has steadily risen to £7M (9.6%) in 2020–2021, £8.5M (11.3%) in 2021–2022 and £10.2M (12.9%) in 2022–2023 (NICE Annual Reports and Accounts 2019–2023). A fee‐for–service drug‐appraisal scheme, brought into existence by Government funding restrictions, has enforced a marketisation of NICE making the agency partly dependent on funding from the pharmaceutical industry to conduct its regulatory work. Doubling such dependence in just 4 years, most of NICE's income could be derived from the commercial industry it is supposed to be regulating in just 10 years’ time.

## 8 Discussion and Conclusion

The extent of revolving doors and conflicts of interests between NICE personnel (including specialist/expert advisors) and the pharmaceutical industry is consistent with both capture theory and corporate bias theory. However, there is considerable evidence that the close relationship between the agency and industry personnel obtained from the inception of NICE. Furthermore, in addition to industry influence on the NICE bureaucracy, there is clear evidence of the pharmaceutical industry being afforded exclusive and privileged access to the executive arm of the Government about NICE's work and policy over and above all other stakeholders (e.g. PICTF, MISG and the NICE‐ABPI Industry Council). While this could imply that some capture has taken place, it is rather inconsistent with capture theory's idea that the agency began life ambitiously adversarial towards, or even entirely independent of, the pharmaceutical industry with a determination to prioritise the public interest over and above those of the industry. Such early partnership between the industry and NICE (albeit sometimes involving NICE's relationship with the executive) is consistent with corporate bias theory. More indirectly, and also consistent with corporate bias, the legislative arm of the UK Government has also been found to have significant financial interests in the pharmaceutical industry via payments to 11% of health‐related All‐party parliamentary groups of MPs between 2012 and 2018 (Rickard and Ozieranski 2021).

Nonetheless, the likely corporate bias of NICE needs to be put in perspective. It is neither as deep nor extensive as Abraham (1995) found regarding the emergence and early decades of the UK drug product regulatory agency and its expert advisors during the 1960s, 1970s and 1980s (e.g. the Committee on Safety of Drugs, the DoH's Medicines Division and the Committee on Safety of Medicines). For example, those regulatory bodies were governed by the 1911 Official Secrets Act and an additional special secrecy clause 118 of the 1968 Medicines Act, which threatened regulators and their advisors with prison if they engaged in any unauthorised public disclosure of information about UK pharmaceutical regulation. That system was so secretive that declarations of financial conflicts of interest of expert regulatory advisors vis‐à‐vis pharmaceutical firms were not even published until 1989 when they were revealed to be more extensive than those at NICE (Abraham and Sheppard 1999). By contrast, NICE has published declared conflicts of interest from the outset.

We are aware of media coverage of cases in which NICE's initial negative decisions have been challenged publicly and in UK courts by alliances of manufacturers and patients/patient organisations. For example, in relation to beta interferon to treat multiple sclerosis, Herceptin for treatment of early‐stage breast cancer, and four drugs (Aricept, Exelon, Reminyl and Ebixa) to treat early‐stage Alzheimer's disease (BBC 2007; Berg 2006; Crinson 2004). These high‐profile cases may have created the impression that NICE took an adversarial position towards the pharmaceutical industry, but overall evidence suggests that these are somewhat exceptional. Moreover, in all these cases, either NICE modified its guidance or the DoH ensured one way or another that the industry and patients gained access to these drugs via the NHS.

NICE has also engaged with a range of stakeholders beyond the pharmaceutical industry, such as NHS medical professionals and patient organisations (e.g. The Partners Council and NICE Citizens Council). Such pluralist tendencies (including wide appraisal consultation and considerable public transparency) have been much more developed than in UK pharmaceutical product regulation, but the extent of privileged influence on NICE by the drug industry leads us to conclude that meso‐level UK pharmaceutical cost‐effectiveness regulation is not fundamentally characterised by pluralism, though it exhibits some pluralist elements.

It could be argued that the influential roles of PICTF, MISG, the Partners Council, the Citizens Council and the law courts imply a de‐centred regulatory system such that NICE should be regarded as a polycentric regulatory agency. However, our conclusion is that such a formulation gives too much weight to a small number of controversial court cases and not enough consideration to revolving doors, conflicts of interest and other evidence of exclusive industry‐NICE partnership, which lead us to conclude that corporate bias is likely to be a better characterisation. Moreover, other research has shown that many entities to which the regulatory agency might be de‐centred, such as expert medical opinion leaders, patient organisations and political elites, are themselves sometimes intertwined with the interests of the pharmaceutical industry (Ozieranski, McKee, and King 2012).

Recent developments at NICE have a distinctly neoliberal flavour. Most notably, permitting pharmaceutical manufacturers to increasingly define evidence‐bases and timelines upon which cost‐effectiveness assessments will be made, together with increasingly allowing regulated pharmaceutical firms to fund the regulatory work to be done on their products. The Thatcher Government's neoliberal reforms of 1989 introduced industry‐fee funding of the UK pharmaceutical product regulatory body, the Medicines Control Agency (MHRA's predecessor), to which the industry agreed in exchange for an accelerated regulatory review system (Abraham and Lewis 2000). Thirty‐two years later, the MHRA's pharmaceutical product regulation was 86% funded by industry fees (Demasi 2022). It remains to be seen whether NICE will travel a similar neoliberal path to that of the MHRA, but there is already sufficient evidence to conclude that ‘neoliberal corporate bias’ is probably a better characterisation of UK pharmaceutical cost‐effectiveness regulation than simply ‘corporate bias’. Indeed, in 2015, even before the introduction of industry fee‐paying, a senior member of NICE's Scientific Advice Team told an open meeting comprising mostly industry attendees that ‘we see the industry as our clients’^8^ (also see Longson, Crabb, and Osipenko 2014). It cannot be known whether NHS pharmaceutical expenditure would have been significantly different in the absence of NICE. Nonetheless, our political‐sociology analysis may go some way to explain why the NHS pharmaceutical bill *rose* annually by over £1 billion even after the establishment of NICE.

## Author Contributions

**John Abraham:** conceptualisation (equal), data curation (equal), formal analysis (equal), funding acquisition (equal), investigation (supporting), methodology (supporting), project administration (equal), supervision (lead), validation (equal), visualisation (equal), writing–original draft (equal), writing–review & editing (lead). **Gowree Balendran:** conceptualisation (equal), data curation (equal), formal analysis (equal), funding acquisition (equal), investigation (lead), methodology (lead), project administration (equal), resources (lead), software (lead), supervision (supporting), validation (equal), visualisation (equal), writing–original draft (equal), writing–review & editing (supporting).

## Ethics Statement

Ethical approval was obtained for this research from King's College London research ethics committee (approval code: KCL MR/15/16‐14).

## Consent

The authors have nothing to report.

## Conflicts of Interest

The authors declare no conflicts of interest.

## Permission to Reproduce Materials From Other Sources

The authors have nothing to report.

## Data Availability

Data are available, some publicly on websites.

## Endnotes

^1^
If there are no alternative health interventions available, then the question is whether the new drug brings sufficient health returns in relation to its costs.

^2^
However, our research here is part of a larger 7‐year project (2014–2021), together with subsequent updates (2021–2024), involving macro‐political concerns and micro‐sociological analysis of NICE decision‐making about case‐study drugs.

^3^
As part of Balendran's Ph.D. research.

^4^
Interview, former special advisor in Policy Unit, 10 Downing Street, 13 May 2019.

^5^
Fieldwork conversation, member of NICE Board of Directors, Liverpool, 14 October 2015

^6^
Fieldwork conversation, senior employee at NICE, Liverpool, 14 October 2015.

^7^
The October 2009 start date was also chosen because that was when NICE began to permit pharmaceutical manufacturers to nominate clinical specialists, enabling such conflicts of interest to be studied. The December 2015 end date was selected because it coincided with the completion of NICE's decision‐making about some case‐study drugs within our larger project.

^8^
Fieldwork observation, ‘Health Technology Assessment as part of Regulatory Strategy?’ NICE‐HTA Conference, London, 27–8 January 2015.
